# Potential use of *Ulva intestinalis*-derived biochar adsorbing phosphate ions in the cultivation of winter wheat *Tristicum aestivum*

**DOI:** 10.1186/s40643-024-00741-z

**Published:** 2024-03-04

**Authors:** Natalia Niedzbała, Ewa Lorenc-Grabowska, Piotr Rutkowski, Jacek Chęcmanowski, Anna Szymczycha-Madeja, Maja Wełna, Izabela Michalak

**Affiliations:** 1 Department of Advanced Material Technologies, Faculty of Chemistry, Wrocław University of Science and Technology, Wrocław, Poland; 2 Department of Process Engineering and Technology of Polymer and Carbon Materials, Faculty of Chemistry, Wrocław University of Science and Technology, Wrocław, Poland; 3 Department of Analytical Chemistry and Chemical Metallurgy, Faculty of Chemistry, Wrocław University of Science and Technology, Wrocław, Poland

**Keywords:** Green seaweed, Pyrolysis, Biochar, Phosphate ions, Wastewater treatment, Soil additives

## Abstract

In this work, the properties of biochar produced from green macroalga *Ulva intestinalis* by pyrolysis were studied at temperatures of 300, 500, and 700 °C. This biochar was characterized in terms of multielemental composition, BET surface area, total pore volume, and biosorption properties toward phosphate ions. Biochar produced at 700 °C–25 m^2^/g had the highest surface area. The kinetics and isotherms of sorption processes of phosphate ions as sorbate by these sorbents were investigated. Modified biochar was able to remove 84.3% of phosphate ions from wastewater, whereas non-modified biochar—only 40.6%. Hence, biochar enriched with phosphate ions can serve as a valuable soil amendment. Pot experiments performed on winter wheat (*Triticum aestivum*) with a 3% addition of dry *Ulva intestinalis*, pristine biochar, and Mg-modified biochar enriched with phosphate ions showed that these amendments stimulated plant growth (length and fresh weight of plants) as well as enlarging the chlorophyll content in leaves. Our results indicate that the production of biochar (pristine and Mg-impregnated) is a sustainable option to valorize the biomass of seaweeds, and to recycle phosphorus from wastewater.

**Graphical Abstract:**

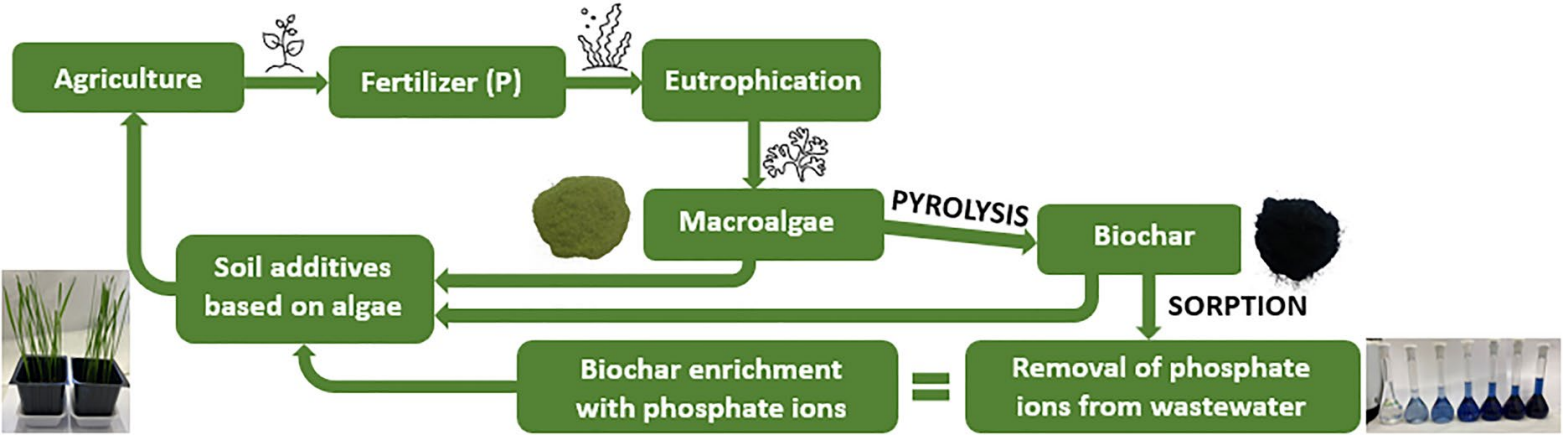

## Introduction

Intense or unmanaged fertilization increases the amount of phosphorus in soils, which when combined with rainfall, causes phosphorus to end up in water bodies (lakes, ponds, rivers, seas, etc.) or sewage (Antunes et al. 2022; Eduah et al. 2020). Eutrophication of water reservoirs increases algae bloom (Arumugam et al. 2018; Jiang et al. 2019). On one hand, these plants are essential for the ecosystem, because they help maintain the aquatic environment in a properly balanced state and provide 50% of the oxygen that sustains life on Earth (Chatzissavvidis and Therios 2014; Michalak et al. 2019). On the other hand, an excess of macroalgae (produced by eutrophication) has a detrimental effect on the environment, in that it damages coral reef health decreases, water clarity, and raises the incidence of fish diseases (Arumugam et al. 2018; Qiu et al. 2019). For this reason, waste biomass that causes environmental nuisance needs to be properly dealt with.

Current research focuses on *Ulva intestinalis*, a green macroalga that has found several applications, for example, in agriculture (use of algae extracts as liquid fertilizers (Chatzissavvidis and Therios 2014), as for animal feed (Oucif et al. 2020), as food for humans (Ganesan et al. 2014), in wastewater treatment (Michalak and Chojnacka 2010) and recently for biochar production (Antunes et al. 2022). The solution mentioned last seems to be of particular interest. Here, algae waste biomass can serve as a raw material in many thermal processes accompanying the production of valuable bioproducts.

Pyrolysis is one of the thermal processes that converts waste and biowaste into such usable products as biochar, bio-oil, or biogas. Pyrolysis precludes the presence of oxygen. The conditions of this thermal process (temperature, time, use of catalysts, etc.) and the type of raw material (from which the biochar will be produced) are very important, because they determine process durability, aromaticity, element content, and the possibility of binding bioproducts with other compounds (Antunes et al. 2022; Leng and Huang 2018; Michalak et al. 2019; Vijayaraghavan and Balasubramanian 2021). Due to their porous structure and their many functional groups, biochars are of great interest as agents removing pollutants from aqueous solutions (for wastewater treatment) and could play an important role in mitigating non-point pollutants (Pinto et al. 2019). Unfortunately, the alkaline pH of biochar and the presence of oxidizing groups that predominate on its surface mean that its ability to bind metal anions such as phosphorus from an aqueous solution is limited (Nardis et al. 2021). To remove selected metal ions, modification/impregnation of biomass is applied before the pyrolysis process or after biochar is produced (Li et al. 2017). Positive sorption results were produced after modifying the raw material before pyrolysis or the biochar with compounds containing cations, for example, magnesium oxide (Li et al. 2017), magnesium chloride (Jiang et al. 2019; Nardis et al. 2021; Pinto et al. 2019; Zheng et al. 2020), aluminium chloride (Zheng et al. 2020), ferric nitrate nonahydrate (Zhang et al. 2021).

Biochar after phosphate ions removal from wastewater can be reused as a soil amendment. Biochar enriched with phosphate ions enhances the physicochemical properties of the soil (by increasing the soil's water content and cation exchange capacity), which makes it easier for plants to access nutrients in the soil and absorb them (Ding et al. 2021; Olmo et al. 2014).

The aim of the present study was to produce biochar from waste algal biomass—*Ulva intestinalis*—collected from the beach of the Baltic Sea, by pyrolysis at 300, 500, and 700 °C. After modification of the seaweed with magnesium chloride, the obtained biochar was examined as a biosorbent of phosphate ions. The utilitarian properties of the pristine biochar and biochar enriched with phosphate ions were examined in phytotoxkit and pot experiments. The idea of this research follows the concept of the circular (bio-based) economy, in which waste algae biomass is processed into biochar used to remove phosphate ions from wastewater, which can then be returned to the soil as an additive to improve its properties and also serve as a source of phosphorus for plants.

## Materials and methods

### Chemicals

The algal biomass before pyrolysis was modified by magnesium (II) chloride (I) hexahydrate (Chempur, Piekary Śląskie, Poland). Potassium dihydrogen phosphate (Eurochem BGD, Tarnów, Poland) served as a source of phosphate ions in the biosorption process. To determine spectrophotometrically the concentration of phosphate ions in an aqueous solution, reagents such as antimony potassium tartrate (Eurochem BGD, Tarnów, Poland), ammonium molybdate (Eurochem BGD, Tarnów, Poland), l( +)-ascorbic acid and sulfuric (VI) acid (Avantor Performance Materials Poland S.A., Gliwice, Poland) were applied. The pH of the solutions was adjusted with the sodium hydroxide and hydrochloric acid (Avantor Performance Materials Poland S.A., Gliwice, Poland). 65% (m/v) nitric acid (Merck, Darmstadt, Germany) was used for microwave-assisted wet digestion of the samples prior to multielement analysis by ICP–OES technique. Multielement ICP standard solution (No. IV) along with single element standards of As, Hg, P, and S (all from Merck, Darmstadt, Germany) calibrated the ICP–OES spectrometer.

### Seaweed biomass

The biomass of green seaweed (S)—*Ulva intestinalis* (syn. *Enteromorpha intestinalis*)—was collected from the beach in Kołobrzeg (Poland) in June 2021. This study did not involve endangered or protected species. Seaweed was identified on the basis of available limnological publications (Dhargalkar and Kavlekar 2004; Messyasz and Rybak 2008). To obtain a homogeneous sample, the biomass was washed with tap water, air-dried, milled in a Retsch GM 300 mill (Retsch Poland, Katowice, Poland) (4000 rpm/min), and sieved (500 μm sieve, Retsch).

The chemical properties of seaweed (related to increased biosorption properties) were modified with the aid of the methodology described by Nardis et al. (2021), i.e., by maceration of this biomass in MgCl_2_∙6H_2_O solution (abbreviated as SMgCl_2_). A solution of 2.67 mol/L magnesium chloride was added to 20 g of *Ulva intestinalis* biomass and stirred for 24 h at 150 rpm (260 basis, IKA^®^ KS, Staufen, Germany), and then dried at 80 °C (SLN 53 SIMPLE, POL–EKO–APARATURA, Wodzisław Śląski, Poland).

### Seaweed pyrolysis

The pyrolysis of seaweed (S) and seaweed modified with MgCl_2_ (SMgCl_2_) was carried out in a Carbolite electric heated furnace (Carbolite Gero, Neuhausen, Germany). A 15 g sample portion was heated in a fixed bed at 300, 500, and 700 °C at 10 °C/min in flowing nitrogen. The final temperature was maintained for 60 min. This produced three biochars: B300, B500, and B700. For seaweed loaded with MgCl_2_, additional biochar at 700 °C (B700MgCl_2_). For this pyrolysis temperature, the best biosorption properties of biochar from seaweed biochar (B700) towards phosphate ions were obtained (results presented later in the article).

### Characteristics of seaweed and biochar

#### Proximate analysis

Analyses complied with the PN–ISO standards. The sample used for the proximate analysis was ground to pass through a sieve with 0.2 mm aperture.

*Moisture determination (PN–EN–ISO 18134-2:2017-03 standard)*. The 1.0 ± 0.1 g sample portion was heated at 105–110 °C in an electric oven. The percentage mass fraction of moisture (*M*_ad_) was calculated from the loss in the test portion.

*Ash determination (PN–ISO 1171 standard)*. The 1.0 ± 0.1 g sample portion was heated at a specific rate up to the temperature of 815 ± 10 °C in an air atmosphere, in a muffle furnace, until a constant mass was obtained. The amount of ash (*A*_ad_) was calculated on the basis of the mass of the residue after incineration.

*Volatile matter determination (PN–EN ISO 18123:2016-01 standard)*. The 1.0 ± 0.1 g sample portion was heated out of contact with air at 90 °C for 7 min in a muffle furnace. The percentage mass fraction of volatile matter (*V*_ad_) was calculated from the mass loss of the test portion after deducting the loss of mass due to moisture. The result, expressed as a percentage by mass, was calculated on a dry, ash free basis according to the following equation:

1 $${\text{V}}_{{{\text{daf}}}} = {\text{V}}_{{{\text{ad}}}} \cdot \frac{100}{{100 - \left( {{\text{M}}_{{{\text{ad}}}} + {\text{A}}_{{{\text{ad}}}} } \right)}}$$

where *M*_ad_ is the moisture in the air-dried coal sample, by mass; *A*_ad_ is the ash of the air-dried coal sample, in mass percent.

*Fixed carbon calculation*. Fixed carbon (*C*_fix, ad_), expressed as a percentage by mass, was calculated on an air-dry basis, from the following equation:

2 $$C_{\rm fix,ad}=100-(M_{\rm ad}+A_{\rm ad}+V_{\rm ad})$$

#### Ultimate analysis

Elemental analysis of C, H, N, S, and O (by difference) was carried out on a Vario III Elemental Analyzer (Langenselbold, Germany). In the measurement, a 5 mg sample was used.

#### Porosity determination

The porous texture was determined from nitrogen adsorption isotherms measured at 77 K with a NOVA 2200 (Quantachrome, Graz, Austria). The specific surface area was calculated using the BET method at *p*/*p*_0_ < 0.30. The amount of nitrogen adsorbed at a relative pressure of *p*/*p*_0_ = 0.99 determined the total pore volume (*V*_T_). The pore volume distribution, bulk density, and porosity were evaluated with a mercury porosimeter (Micromertics AutoPore IV 9510, Micromeritics Instrument CORP, Norcross, USA). Operating pressures ranged between 0.1 and 400 MPa. Before measurement, the samples were degassed under vacuum at 300 °C.

#### Measurement of pH and electrical conductivity

The methodology described by Cantrell et al. (2012) was used to measure the pH and electrical conductivity. For this purpose, a 1% aqueous solution of seaweed/biochar was prepared. The suspension was shaken (100 rpm, 2 h, 260 basic, IKA^®^ KS, Staufen, Germany), filtered and finally measured (SevenCompact Duo, Mettler-Toledo, Ohio, USA). Samples were analyzed in two parallel replicates (*N* = 2).

#### Fourier transform infrared spectroscopy

The analysis of functional groups on the surface of the samples tested was carried out using a spectrometer (Vertex 70 V, Brucker, Massachusetts, USA) equipped with an air-cooled DTGS detector under vacuum conditions. The weakened internal reflection technique was carried out with a diamond crystal single reflection ATR attachment. The spectra had the range of 4000–400 cm^−1^, where 64 scans with a resolution of 4 cm^−1^ were performed.

#### Scanning electron microscopy

Biochar surface examination was carried out using scanning electron microscope with EDS system (Quanta 250 LEICA EM ACE200, FEI, Oregano, USA). Vacuum sputtering was employed to cover non-conductive samples with a thin layer of gold (approximately 10 nm thick). Samples were examined in duplicate replicates (*N* = 2).

### Biosorption studies of phosphate ions by biosorbents

The influence of the initial pH of the solution containing phosphate ions on their biosorption. The influence of the initial pH of the solution with phosphate ions on the biosorption process was checked first. For this purpose, solutions containing phosphate ions were prepared at a concentration of 100 mg/L with an appropriate pH value (2–8). 0.02 g of sorbent (seaweed/biochar) was added to 20 mL of the solution with a given pH and left for 4 h. After this time, the suspension was filtered. The pH of the solution after biosorption was then measured, and the phosphate ions were determined using the colorimetric method.

#### Kinetics of biosorption

The sorption process of phosphate ions was carried out on biochar (B300, B500, B700) and modified biochar produced at a temperature of 700 °C (B700MgCl_2_) according to the methodology described by Michalak and Chojnacka (2010) and Papirio et al. (2017). The initial solution contained phosphate ions at a concentration of 100 mg/L and a pH of 5 or 6 (based on the previous stage described). 0.200 g of biochar was weighed in an Erlenmeyer flask and 200 mL of the appropriate solution was added. The samples were shaken (225–230 rpm) and 10 mL was collected at appropriate intervals (5, 10, 15, 30, 45, 60, 90, 120, 180, 240 min). Subsequently, the solutions were filtered through filter paper. The filtrates were used to determine the concentration of phosphate ions.

#### Equilibrium of biosorption

The equilibrium of the sorption process by modified biochar (B700MgCl_2_) was performed according to the methodology developed by Michalak and Chojnacka (2010). Initial solutions containing phosphate ions were prepared at concentrations of 5, 10, 25, 50, 75, 100 mg/L and the pH of each was adjusted to 5. The sorbent content in the solution was 1 g/L or 10 g/L (this concentration was used to enrich biochar with phosphate ions for later tests on plants). After shaking for 3 h at 100 rpm, 10 mL was taken and filtered through filter paper. After biosorption, the pH of the solutions and the concentration of phosphate ions were measured with using appropriate analytical methods. The biochar enriched with phosphate ions was air dried at room temperature and applied in subsequent pot experiments. Each biosorption process was analyzed in duplicate (*N* = 2).

#### Biosorption process modelling

The sorption process is described by means of various kinetic models that explain the mechanism of this process. The two most common kinetics models are the pseudo-second-order (PSO) model and the pseudo-first-order (PFO) model, which assume that the sorption rate is proportional to the availability of the free spaces on the sorbent surface. Consequently, they are used in the theoretical explanation of adsorption at the solution/solid interface. These models enable the determination of two important parameters, the equilibrium sorption capacity, and the constant speed of the sorption process (Ho et al. 1996). The PFO model is described by the following equation:

3 $$\frac{{{\text{d}}q_{t} }}{{{\text{d}}_{t} }} = k_{1} \cdot \left( {q_{{{\text{eq}}}} - q_{t} } \right)$$

The linear form of the equation for PFO after taking into account the boundary conditions [i.e., *q*(*t*) = 0] is described as the following equation:

4 $$\ln \left( {q_{{{\text{eq}}}} - q_{t} } \right) = \ln q_{{{\text{eq}}}} - k_{1} \cdot t$$

Equation (5) describes the pseudo-second-order model:

5 $$\frac{{{\text{d}}q_{t} }}{{{\text{d}}t}} = k_{2} \cdot (q_{{{\text{eq}}}} - q_{t} )^{2}$$

After linearization and with the consideration of the boundary conditions, it is expressed as the following equation:

6 $$\frac{t}{{q_{t} }} = \frac{1}{{\left( {k_{2} \cdot q_{{{\text{eq}}}}^{{{ }2}} } \right)}} + \frac{t}{{q_{{{\text{eq}}}} }}$$

where *q*_eq_ is the equilibrium sorption capacity of a sorbent (mg/g); *q*_t_ is the sorption capacity in given time *t* (mg/g); *t* is time (min); *k*_1_ is the rate constant of the PFO model (1/min); *k*_2_ is the rate constant of the PSO model [g/(mg·min)].

The dynamics of the sorption process is described with using a variety of diffusion models. They are based on the observation that the rate of biosorption is limited by the molecular diffusion (at the interface, inside the sorbent, with the active site of the sorbent). The Weber–Morris model describes intraparticle diffusion and the determination of the diffusion rate constant (*k*_id_); also, the thickness of the boundary layer (*C*) can be calculated from the following equation (Jha et al. 2019; Weber and Morris 1963):

7 $$q_{{\text{t}}} = k_{{{\text{id}}}} \cdot t^{\frac{1}{2}} + C$$

The isotherm of the sorption process refers to the relationship between the concentration of sorbate and sorbent, i.e., between a solid and a liquid containing sorbate ions. The most frequently used isotherm to describe the equilibrium of the sorption process is the Langmuir isotherm (Langmuir 1918). It makes it possible to obtain information about the adsorption mechanism and the maximum sorption capacity of a given sorbent with respect to sorbate (Ayawei et al. 2017). Equation (8) takes the form of:

8 $$q_{{{\text{eq}}}} = q_{{{\text{max}}}} \cdot \frac{{b \cdot C_{{\text{e}}} }}{{1 + b \cdot C_{{\text{e}}} }}$$

where *q*_max_ is the maximum sorption capacity (mg/g); *q*_eq_ is the equilibrium sorption capacity (mg/g); *b* is the Langmuir constant (L/mg); *C*_e_ is the equilibrium concentration of the adsorbate (mg/L). The separation factor *R*_L_, a dimensionless constant, can be used to determine the fundamental properties of the Langmuir isotherm, following:

9 $$R_{{\text{L}}} = \frac{1}{{1 + b \cdot C_{0} }}$$

where *C*_0_ is the initial concentration of adsorbate (mg/g).

According to the *R*_L_ value, adsorption is either irreversible (*R*_L_ = 0), favorable (0 < *R*_L_ < 1), linear (*R*_L_ = 1), and unfavorable (*R*_L_ > 1) (Ayawei et al. 2017; Michalak and Chojnacka 2010).

Another Eq. (10) frequently used to describe the sorption of metal ions is the Freundlich isotherm model (Freundlich 1907):

10 $$q_{{{\text{eq}}}} = k_{{\text{F}}} \cdot C_{{\text{e}}}^{\frac{1}{n}}$$

where *k*_F_ is Freundlich affinity coefficient; *n* is Freundlich linearity constant.

### Seaweed and biochar as a soil additive

To investigate the potential phytotoxicity of *Ulva intestinalis* and derived biochar on plants, phytotoxkit (TK61, Tigret Sp. z o.o., Warsaw, Poland) and pot experiments were conducted on winter wheat (*Triticum aestivum*). For the tests, commercial soil (Athena “universal potting soil”, Szczecinek, Poland) was used. According to producer information, the pH of the peat-based soil, enriched with micro- and macroelements, was in the range of 5.5–6.5. Fresh soil was dried and subjected to sieve analysis (2 mm sieve). The soil was evenly mixed with 3% seaweed or seaweed-derived biochars and left for two weeks. For the phytotoxkit tests, the following soil additives were applied: *Ulva intestinalis* seaweed, biochar produced at temperatures 300, 500, and 700 °C. Each phytotoxkit test contained 10 seeds, which were placed at equal intervals. Nine seeds were included in the pot experiments. The prepared tests were placed vertically under a 45W LED Grow Light (GL-225RB-45W) at a height of 57 cm, with a 12/12 h photoperiod (ST 5C SMART, POL–EKO–APARATURA, Wodzisław Śląski, Poland). All phytotoxkit tests lasted 8 days.

In the pot experiments, biochar (B700) and modified biochar obtained at 700 °C (B700MgCl_2_) enriched with phosphate ions (B700 + P and B700MgCl_2_ + P, respectively) in the biosorption process were examined. The content of these additives in pots with soil was equal to 3%. The potted wheat was watered three times a week and the whole pot experiments lasted for 2 weeks. Both tests were performed in triplicate (*N* = 3).

The length of the aboveground parts of the plants and the chlorophyll content in the leaves were then measured. Finally, the fresh weight of the aboveground parts was weighed, and the pH of the soil was measured (SevenCompact Duo, Mettler-Toledo, Ohio, USA) for each of the plates and pots. For the pot experiments, the leaf area was measured using WinFOLIA Regular Software Program. The relative chlorophyll content was also measured in plant leaves with a SPAD device (SPAD 502 Chrolophyll Meter, Spectrum Technologies, Inc., Aurora, USA). The values indicated by the meter are proportional to the chlorophyll content in the leaf. They were calculated on the basis of the amount of radiation transmitted through the leaf in two radiation ranges, differently absorbed by chlorophyll.

### Analytical methods

#### Colorimetric method

The colorimetric method according to the PN–EN ISO 6878:2006 standard determined phosphate ions in solutions before and after biosorption. To measure the content of phosphate ions, water and 2.0 mL of sample solution were transferred to the volumetric flask (50 mL) and mixed with 1.0 mL of 10% (m/v) aqueous ascorbic acid solution, 2.0 mL of colored reagent [which it consisted of 100 mL of 13% solution of ammonium molybdate, 100 mL of 0.35% solution of antimony potassium tartrate, 150 mL of 65% sulfuric(VI) acid and 150 mL of water in ratio 1:1] and then made up to 50 mL with distilled water. After 10 min, the solution became dark blue and the absorbance was measured on a spectrophotometer (Biosens UV 5100, Metash, Shanghai, China) at 700 nm. Quantification was performed against an external calibration within the analyte concentration range of 2–100 mg/L.

#### Multielement analysis by spectrometric method

Measurements of concentrations of elements in the tested samples (a dry seaweed sample, modified with MgCl_2_ seaweed, biochars (produced at 300, 50, 700 °C), and modified biochar) were made using an inductively coupled plasma-optical emission spectrometer with dual view Ar-ICP (model 5100 Dual View, Agilent Technologies Inc., Santa Clara, California, USA), against solutions of simple standards. Operating instrument settings, recommended by the manufacturer, were applied. Before analysis, the samples were digested in a microwave-assisted closed-vessel system (Multiwave PRO, Anton Paar, Graz, Austria). In the procedure, samples (0.25 g) were weighed into PTFE vessels and poured with 5.0 mL of 65% (m/v) HNO_3_. Vessels were closed, inserted into the rotor and subjected to the microwave-assisted heating program with a maximum temperature of 190 °C for 60 min. The sample digests were quantitatively transferred into 30 mL containers and made up with deionized water to 25.0 g. Then, the resulting solutions were filtered through 0.45 µm membrane syringe filers. Simultaneously, blank samples were prepared and included in the final results. All samples’ solutions were prepared by weight (to avoid differences in their density) and analyzed in triplicate (*N* = 3).

#### Statistical analysis

Statistica version 13.0 (TIBCO Software Inc., Tulsa, USA) served the purpose of statistically analyzing the results. Descriptive statistics was carried out for each experimental group. The Shapiro–Wilk test verified the normality of the distribution of test results, while the Brown–Forsythe test examined the homogeneity of variances. The results led to the selection of the statistical test (used to determine the statistically significant differences between the experimental group). The statistically significant differences among the groups could be identified with the one-way analysis of variance (ANOVA). If the assumption of normal distribution and homogeneous variances was correct, the Tukey multiple comparison test was applied. Otherwise, the Kruskal–Wallis test was performed to determine statistically significant differences for *p* < 0.05.

## Results and discussion

### Production of biochar

The pyrolysis yield for the temperature 300 °C was 62%, for 500 °C–53%, for 700 °C–43% and for pyrolysis of *Ulva intestinalis* loaded with Mg(II) ions at 700 °C it was 37%. The yield of biochar produced from the algae decreased as pyrolysis temperature increased. It should be noted that the efficiency of pyrolysis processes is relatively high. Higher temperature leads to a greater decomposition of the organic part of algae, which this is consistent with other studies—for freshwater macroalga *Cladophora glomerata*, the pyrolysis yield was 63% for 300 °C, 56% for 350 °C, 50% for 400 °C, and 47% for 450 °C (Michalak et al. 2019). Also, Antunes et al. (2022) noted a relationship between an increase in pyrolysis temperature and a decrease in the process efficiency. In the cited work, the yield of biochar produced from macroalgae *Ulva ohnoi* at 300 °C was 60.4% and at 800 °C was 29.4%. The high yield of algal biochar is due to its high ash content (Table 1). The lowest pyrolysis yield is observed for B700MgCl_2_. This is due to the fact that modified biochar with MgCl_2_ also decomposes at a temperature higher than 300 °C.

**Table 1:** Proximate and ultimate analysis of dry seaweed and seaweed-derived biochar

Sample	Proximate analysis				Ultimate analysis			
	*M*_ad_	*A*_ad_	*V*_daf_	*C*_fix,ad_	C	H	N	S
	wt%, dry basis							
S	6.1	20.1	89.0	7.8	23.5	3.94	2.36	2.68
SMgCl_2_	10.2	28.0	98.7	0.8	7.73	6.24	0.70	1.31
B300	4.3	48.3	71.3	13.6	34.3	3.02	3.40	4.33
B500	4.9	55.2	44.5	22.1	31.5	1.46	2.80	4.62
B700	4.9	69.3	25.2	19.3	32.5	0.49	2.24	5.03
B700MgCl_2_	10.0	62.0	31.3	19.2	15.8	1.72	0.71	1.36

### The preliminary techno-economic aspect of biochar production

In this article, biochar was produced from green macroalga *Ulva intestinalis*, a waste raw material, which in turn means that its acquisition costs were zero. However, the seaweed was collected at the seaside, which is why there are costs of transporting the raw material to the place of biochar production. Preparation of algae for the pyrolysis process consisted of air drying (no costs), grinding it in a grinder (which generates very low electricity costs), and sieve analysis (no costs). Pyrolysis used to obtain biochar is the most expensive stage, because a large amount of electricity is needed to initiate this process. However, by using the remaining fractions (e.g., by selling them), this allows to cover the costs associated with producing biochar (Kumar et al. 2020). Carrying out the modifications on biomass and producing the modified biochar will generate higher production costs compared to unmodified biochar due to the lower efficiency of the pyrolysis process (37%) and the need to use a reagent (MgCl_2_) and additional equipment (shaker).

In the article by Antunes et al. (2022) carried out a techno-economic analysis of biochar obtained from macroalgae *Ulva ohnoi* at a temperature 700 °C, which showed that the cost of biochar production was USD 710/tonne. It was also calculated that it was possible to reduce costs to USD 485/tonne by selling the remaining pyrolysis products (bio-oil and biogas) produced. Antunes et al. (2022) emphasized that the resulting high-quality biochar from macroalgae compensates for the costs of its production due to its phosphorus adsorption ability.

### Characteristics of seaweed biochar

#### Proximate and ultimate analysis of seaweed and seaweed-derived biochar

The proximate and ultimate analysis of seaweed and seaweed-derived biochar are presented in Table 1.

The moisture content of the fresh algal biomass is considerably high (> 85%) (Farzanah et al. 2022). In this work, however, it was relatively low. As can be seen in Table 1, the amount of residual moisture determined in the dry algae ranged from 6.1 wt% in the raw seaweed, through 4.3–4.9 wt% in the heat-treated seaweed (biochar), to more than 10 wt% in the modified seaweed. This led to the conclusion that simple air drying easily removes incidental moisture, while the residual moisture found in this material is mainly due to the type of mineral matter present in the algae. This was evident when the moisture content of raw seaweed and that modified seaweed were compared. The introduction of MgCl_2_ improved hydrophilicity; therefore, the moisture in SMgCl_2_ and B700MgCl_2_ samples was higher than in raw materials. Heat treatment reduced the moisture content in seaweed, biochar, and B700MgCl_2_ series, but the B700MgCl_2_ still had the moisture content close to the SMgCl_2_.

The algal biomass was rich in mineral matter and its content increased significantly with the applied thermal treatment. This was due to the decomposition of the organic matter of the algal biomass during pyrolysis and was expressed by a decrease in the amount of volatile matter (Table 1). The volatile matter for seaweed varies from 45 wt% to 90 wt% (Lee et al. 2020). Therefore, the results obtained in this work were in the upper range of the average results. The fixed carbon content (i.e., a part of the sample that remains after subtracting the moisture, ash, and volatile matter contents) typically had a trend opposite to that of the volatile matter content. It was a measure of the solid combustible material that remains after the volatile matter had been removed. It can be seen that the lowest fixed carbon was found in SMgCl_2_. Thermal treatment enlarged the biochar combustible part of the product and remained at almost the same level after heating at a temperature higher than 500 °C.

The thermal conversion of seaweed caused an increase in carbon content from 23.5% for raw seaweed to 34.3 wt% for B300. The carbon content in the biochar was comparable. Unfortunately, no linear tendency with an increase in pyrolysis temperature was observed. On the contrary, such a relationship was found for the hydrogen content. A linear decrease in its content was coupled with an increase in pyrolysis temperature. The nitrogen content of the algal biochar was slightly higher or comparable to that of raw seaweed, while the sulfur content was almost double. The seaweed loaded with MgCl_2_ had a much lower C, N, S content compared to the seaweed. Chemical modification is a procedure that makes biochar more mineralized and thus reduces its carbon content (Nardis et al. 2021). Similarly, to seaweed, the heat treatment of SMgCl_2_ increased the carbon content and decreased the hydrogen content, while S and N remained unchanged.

#### Porosity determination

**Fig. 1:**
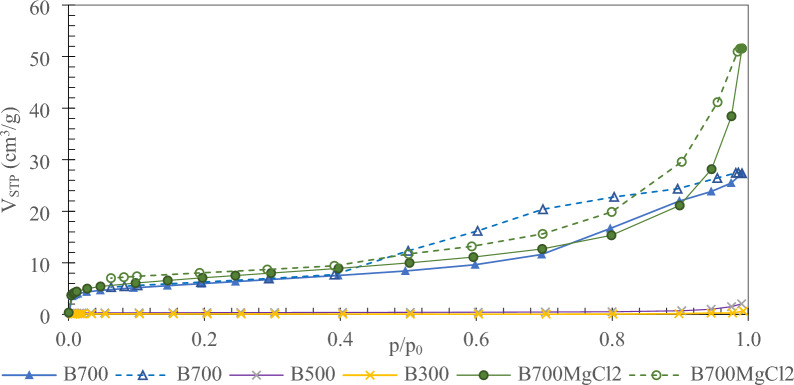
Nitrogen sorption at 77 K on seaweed and biochars

Surface area and porosity are characteristics that play a crucial role in the adsorption process. The raw material from which biochar was produced and the biomass grain size have a significant impact on the porosity and total surface area. The BET surface area for raw seaweed was very small, but yet the pyrolysis process developed the biochar surface area. The *S*_BET_ (specific surface area) increased from 0.3 m^2^/g for B300, through 1.3 m^2^/g for B500, to 25 m^2^/g for B700. The surface area for B700MgCl_2_ was comparable to that of B700 biochar and was 21 m^2^/g. The thermal treatment of seaweed led to a significant development of porosity. The dependence of the increase in specific surface area on the temperature of the pyrolysis process was also found by Antunes et al. (2022) for the pyrolysis of macroalga *Ulva ohnoi* at 800 °C. The hysteresis loop that can be found in the shape of nitrogen sorption isotherm for B300, B500, B700, and B700MgCl_2_ samples indicates that mainly large mesopores and macropores were developed; however, based on the hysteresis shape, a different type of porosity was evolved in biochar B700 and B700MgCl_2_ (Fig. 1). The volume of a nitrogen adsorbed at *p*/*p*_0_ below 0.4 was comparable. A notable volume increase at *p*/*p*_0_ > 0.9 was observed for B700MgCl_2_. The hysteresis loop for B700MgCl_2_ was obtained for adsorbents having more slit-shaped pores (type H3) whereas in the case of B700 the hysteresis loop indicates that the distribution of pore size and shape was not well defined.

The results of the mercury porosity measurement for biochar (Table 2) reveal many similarities to the nitrogen sorption data. The surface area increased with increasing pyrolysis temperature. The highest pore area was observed for B700. The B700MgCl_2_ was also characterized by a high but slightly lower surface area. In turn, it had an almost threefold higher total intrusion volume. This is because the biochar structure changes after biomass modification with magnesium chloride (Li et al. 2017). The sample also had a higher median pore volume and porosity compared to those of raw biochar, indicating different porosity compared to that of raw algal biochar. It can be observed that for the unmodified biochar, the porosity increases with increasing pyrolysis temperature, while the average pore diameter decreases. The average pore diameter for modified biochar was almost four times higher than that of the B700 biochar. The apparent density is closely related to the mass of the sample and the volume occupied by the solid sample, including solid phases, particle pores, and interparticle voids (Brewer et al. 2014; Chia et al. 2015). The apparent density value should increase with the increase in the temperature of the pyrolysis process due to the gradual condensation of carbon, which can be seen for biochars produced at temperatures 500 and 700 °C. However, for biochar obtained at a temperature 300 °C, the apparent density value is the highest among all the analyzed biochars, which is related to the highest carbon content for this biochar, i.e., 34.3%. This correlation between the carbon content in biochars and the apparent density can also be noticed for other analyzed in the study biochars. Attention should be paid to the change in the porous structure that occurs in the samples under investigation as pyrolysis temperature increases. As mentioned above, an increase in pyrolysis temperature leads to the development of a porous structure. Hence the combination of these two factors, (i) the decomposition of the organic part causing a decrease in the C, H, N, S content and (ii) the changes in the porous texture, cause this non-linear change in the apparent density.

**Table 2:** Porous texture from mercury porosimetry

Sample	Total intrusion volume	Total pore area	Apparent (skeletal) density	Median pore diameter (area)	Average pore diameter (4* V*/*A*)	Porosity
	cm^3^/g	m^2^/g	g/cm^3^	nm	nm	%
B300	0.15	5.56	0.554	6.5	108	7.67
B500	0.20	8.72	0.496	6.1	94	9.18
B700	0.19	19.0	0.550	6.4	39	9.29
B700MgCl_2_	0.71	16.5	0.408	19	173	22.6

4* V*/*A* an equation, where *V* (volume) and *A* (area), according to the cylindrical pore

#### pH and conductivity

To determine the alkalinity and susceptibility to the flow of electric current, pH and conductivity were measured in raw seaweed and biochars. It was observed that the increase in pyrolysis temperature increased the alkalinity of biochar (7.96 ± 0.27 for B300, 9.59 ± 0.11 for B500, 10.5 ± 0.1 for B700). Compared to B700, the pH of the biochar that had been modified with MgCl_2_ was slightly lower (9.90 ± 0.11). This is in agreement in line with the results reported by Nardis et al. (2021) for biochar produced at 500 °C from poultry litter and modified with magnesium chloride. Furthermore, similar results were achieved by Cantrell et al. (2012), i.e., for biomass—poultry litter pH was 8.2, for biochar produced at 350 °C pH was 8.7, and for biochar obtained at 700 °C pH was 10.3. In our work, as mentioned above, the biochar obtained at the temperature of 700 °C was the most alkaline, while the lowest pH value was for raw seaweed (6.51 ± 0.06).

The modified biochar produced at a temperature of 700 °C had the highest conductivity value—3202 ± 124 μS/cm. A similar electrical conductivity value (2810 μS/cm) was reported for biochar produced from maize straw at temperature of 350–550 °C in a study by Zhao et al. (2017) compared to B300 and B500 obtained here (2460 ± 20 and 2482 ± 2 μS/cm). The lowest conductivity value was 975 ± 10 μS/cm for seaweed and 1475 ± 14 μS/cm for B700. Vijayaraghavan and Balasubramanian (2021) found that the pH and conductivity of biochar originating from pinewood waste (mulch) increased as pyrolysis temperature increased from 300 to 600 °C. In their research, electrical conductivity values ranged from 32.5 to 51.2 μS/cm. Our results were higher, which may probably be related to the use of other materials in the production of biochar. Due to the presence of sodium chloride in salt water, marine algae had higher electrical conductivity than freshwater algae (Postma et al. 2002). Therefore, biochar from marine alga *Ulva intestinalis* had higher electrical conductivity compared to the biochar used in the cited work (Vijayaraghavan and Balasubramanian 2021).

#### Multielement analysis

The results of multielement analysis of the examined samples (*Ulva intestinalis*, modified *Ulva intestinalis*, biochar made from seaweed at a temperature of 300, 500, and 700 °C, and modified biochar obtained at 700 °C) by ICP–OES are presented in Table 3. Accordingly, it can be concluded that *Ulva intestinalis* was rich in valuable macro- and microelements, for example, K, Mg, S, and P, which play an important role in plant nutrition. Our results for Na and K are similar to those obtained by Oucif et al. (2020) for seaweed *Enteromorpha compressa*, that is, 3.7 g/kg (Na) and 10.8 g/kg (K). Importantly, for *Ulva intestinalis*, tested in this study, some heavy metals (such as Cu, Pb, Cd, As, and Hg) were below their detection limit, which is crucial due to the potential use of these algae and products made from it in agriculture.

**Table 3:** Multielement composition of seaweed and seaweed-derived samples (mean ± SD; mg/kg d.m.)

Element	S	SMgCl_2_	B300	B500	B700	B700MgCl_2_
Al	1150 ± 1	461 ± 4	1914 ± 3	2694 ± 6	3402 ± 21	1441 ± 35
As	< LOD	< LOD	< LOD	< LOD	< LOD	< LOD
B	111 ± 1	46.7 ± 0.2	200 ± 1	233 ± 1	261 ± 1	100 ± 1
Ba	6.72 ± 0.01	3.22 ± 0.08	10.6 ± 0.1	14.5 ± 0.1	19.6 ± 0.1	9.57 ± 0.23
Ca	9730 ± 47	3212 ± 15	14466 ± 25	19136 ± 44	21125 ± 131	7232 ± 95
Cd	< LOD	< LOD	< LOD	< LOD	< LOD	< LOD
Cr	84.8 ± 0.8	45.3 ± 0.3	94.7 ± 0.2	121 ± 1	161 ± 1	105 ± 2
Cu	< LOD	< LOD	< LOD	0.494 ± 0.005	1.87 ± 0.02	< LOD
Fe	1926 ± 6	766 ± 9	2926 ± 25	3790 ± 49	4470 ± 92	1681 ± 31
Hg	< LOD	< LOD	< LOD	< LOD	< LOD	< LOD
K	12768 ± 80	4848 ± 5	22863 ± 421	26174 ± 324	30345 ± 543	14505 ± 580
Mg	156457 ± 2082	92667 ± 569	222273 ± 8984	316711 ± 4346	345958 ± 1209	204971 ± 11145
Mn	115 ± 2	42.2 ± 0.5	188 ± 1	241 ± 1	266 ± 1	98.1 ± 1.8
Na	3469 ± 23	2141 ± 73	5459 ± 9	6771 ± 16	7639 ± 47	4639 ± 123
Ni	38.8 ± 0.2	16.6 ± 0.4	43.1 ± 0.2	54.7 ± 1.3	72.5 ± 1.2	42.3 ± 0.2
P	1319 ± 22	465 ± 12	2022 ± 42	5487 ± 29	2686 ± 55	1055 ± 5
Pb	< LOD	< LOD	< LOD	< LOD	< LOD	< LOD
S	27925 ± 45	10832 ± 181	38970 ± 608	46984 ± 720	43360 ± 1232	12351 ± 206
Zn	14.8 ± 0.5	< LOD	27.3 ± 0.2	34.4 ± 0.1	41.5 ± 0.2	< LOD

< *LOD* below limit of detection; d.m. dry mass

Comparing the values of the elements content in algae with the biochars produced, it can be seen that *Ulva intestinalis* had lower values among all the analyzed elements. In general, the content of all elements in biochar increased with the increase in pyrolysis temperature. The same relationship was observed for the elements: Ca, K, Mg, and P by Michalak et al. (2019), who produced biochar from the freshwater macroalgae *Cladophora glomerata* at four different temperatures (300, 350, 400, and 450 °C). Antunes et al. (2022), who used the macroalga *Ulva ohnoi* to produce biochar at temperatures of 300, 400, 500, 600, 700, and 800 °C, observed a similar trend. As the temperature of biochar production increased, so did the content of all tested elements (Antunes et al. 2022). The content of alkaline elements also increases with increasing pyrolysis temperature, which is why it is suggested as a liming agent to correct the pH of acidified soil (Al-Wabel et al. 2013).

The modification of algal biomass with magnesium chloride and the production of biochar from this biomass also influenced the content of elements, despite the use of the same pyrolysis temperature. For modified biochar produced at 700 °C, the content of elements was lower compared to that of the biochar obtained at 700 °C. Nardis et al. (2021) studied the content of Mg, Ca, and P in biochar (made from poultry litter, pig manure, and sewage sludge at 500 °C) before and after magnesium chloride modification. For Ca and P, closely related to our results, lower values were obtained for the modified biochar. On the contrary, the Mg content in the article by Nardis et al. (2021) was higher for modified biochar. The lower Mg content could be caused by a different structure of the biomass used to produce this biochar.

### Removal of phosphate ions

#### Influence of the initial pH on the biosorption of phosphate ions by sorbents

Prior to the sorption experiments (kinetics and equilibrium), the effect of pH (2–8) of the initial solution containing phosphate ions on the sorption capacity of biochar towards these ions was examined. The following sorbents were considered: *Ulva intestinalis*, biochar produced at various temperatures of 300, 500, and 700 °C, and modified biochar obtained at 700 °C. The results are presented in Table 4.

**Table 4:** Summary of the measured pH values before and after the sorption process of removal phosphate ions and the sorption capacity for seaweed and seaweed-derived samples (*C*_0 _100 mg/L, *C*_s_ 1 g/L, 4 h)

S			B300			B500			B700			B700MgCl_2_		
pH		*q*_eq_ (mg/g)	pH		*q*_eq_ (mg/g)	pH		*q*_eq_ (mg/g)	pH		*q*_eq_ (mg/g)	pH		*q*_eq_ (mg/g)
Before sorption	After sorption		Before sorption	After sorption		Before sorption	After sorption		Before sorption	After sorption		Before sorption	After sorption	
1.98	1.98	No sorption*	2.02	2.60	No sorption*	2.01	2.32	0.873 ± 0.448	2.02	2.82	0.566 ± 0.378	2.04	2.59	10.7 ± 3.68
3.04	3.04		3.00	5.10		2.99	6.07	2.64 ± 1.32	3.01	7.42	7.36 ± 1.32	3.02	6.98	7.63 ± 2.30
4.05	4.05		4.00	5.41		4.03	6.30	2.22 ± 0.94	4.03	8.44	7.31 ± 1.56	4.02	7.07	7.34 ± 2.80
5.01	5.01		5.04	5.69		5.04	6.55	1.06 ± 0.02	5.00	9.16	9.01 ± 1.79	5.03	6.92	8.49 ± 3.29
6.01	6.01		6.01	6.09		5.96	6.65	1.04 ± 0.19	6.02	8.87	6.80 ± 0.66	6.04	6.98	12.9 ± 7.60
7.05	7.05		6.98	6.70		6.99	7.16	2.90 ± 0.87	6.98	9.58	6.16 ± 1.58	6.98	7.30	8.91 ± 6.34
7.96	7.96		7.99	7.06		7.98	8.20	2.60 ± 0.28	8.02	9.89	8.92 ± 0.14	8.03	8.44	12.8 ± 10.2

^*^No sorption (using colorimetric method for phosphate ions determination)

The pH value (2–8) of the solution containing phosphate ions was determined prior to and following the sorption process. Seaweed *Ulva intestinalis* did not cause changes in the pH value after the sorption process. Comparing the pH values of the solutions after the sorption of phosphate ions by three biochars—B300, B500, and B700, it can be noticed that the pH of solution increases with the increase in the temperature of biochar production. This is related to the increased porosity development, alkalinity of biochar (as presented in the pH and conductivity section), and the elimination of acidic functional sites. The alkalinity of biochar increases as the temperature of the pyrolysis process increases (Cantrell et al. 2012; Vijayaraghavan and Balasubramanian 2021). In addition, with the increase in the pyrolysis temperature, the content of alkali and alkaline earth metals (K, Na, Ca, Mg) in biochar increased (increase in the pH of biochar) (Table 3).

Depending on the pH of the initial solution, the phosphate ion removal efficiency of the seaweed/biochar varies. At pH below 3, adsorption is difficult, because the phosphate ions are in the form of H_3_PO_4_. For all solution with pH above 3, a noticeable increase in sorption capacity was observed, which may be due to the alkaline composition of the biochar and the fact that phosphate ions take the form of HPO_4_^2−^ and H_2_PO_4_^−^ (Aryal et al. 2020; Qiu et al. 2019). For *Ulva intestinalis* and biochar produced at 300 °C, the process of sorption of phosphate ions was inefficient. Qiu et al. (2019) found, the same relationship: a higher biochar production temperature (300, 400, 500 °C) increased the amount of adsorbed phosphate ions by biochar (below pH 4, poor or no sorption). The experiments carried out in this study showed that the values of the sorption capacity for B500 ranged from 0.873 to 2.90 mg/g. In the cited work (Qiu et al. 2019), it was shown that for biochar made from *Broussonetia papyrifera* leaves at 500 °C, sorption capacity values did not exceed 8 mg/g. As part of the investigation, it was discovered that biochars produced at a temperature of 700 °C had a higher adsorption capacity compared to that of other biochars. The highest value, which is 9.01 mg/g, was recorded for pH 5. Therefore, B700 was selected for sorption experiments (kinetics and equilibrium), as a sorbent, and the pH of the solution containing phosphate ions was set to 5. In another work (Antunes et al. 2022) the highest sorption capacity with respect to phosphate ions was obtained for biochars produced from green seaweed *Ulva ohnoi* at 700 °C and 800 °C, but for economic reasons, biochar with lower temperature was selected for further research.

The analysis of the influence of solution pH on the sorption of phosphate ions showed that the pH value increased after the sorption process, and the highest sorption capacity was obtained for pH 6 (12.9 mg/g) and 8 (12.8 mg/g). B700 and B700MgCl_2_ were selected for further research as the most promising sorbents of phosphate ions, and to carry out the sorption process under the same experimental conditions, an initial pH of 5 was selected for the solution of these ions. At this pH, the highest sorption capacity of phosphate ions for unmodified biochar was obtained.

#### Kinetics of sorption process of phosphate ions

As mentioned above, biochar B700 was selected to study the sorption kinetics of phosphate ions because of its highest value of sorption capacity among other biochars tested. Based on the above studies, a full adsorption study should be carried out on the adsorbent with the highest porosity and the most positively charged surface, which is why the biochar produced at 700 °C from *Ulva intestinalis* biomass modified with MgCl_2_ was chosen. The choice of MgCl_2_ was intentional due to its non-toxicity and ability to form the cationic bridge, which can significantly increase the adsorption capacity of biochar towards phosphate ions and the potential to be used as a soil amendment (Nardis et al. 2021). The effect of pH (2–8) of the solution containing phosphate ions on the sorption capacity of modified biochar produced at 700 °C (B700MgCl_2_) was examined and the highest value of 12.9 mg/g was obtained for pH 6. For this biochar, an equally high biosorption capacity value was reached for pH 5 (8.49 mg/g), and to standardize the research conditions, pH 5 was taken for further kinetic and equilibrium experiments.

**Fig. 2:**
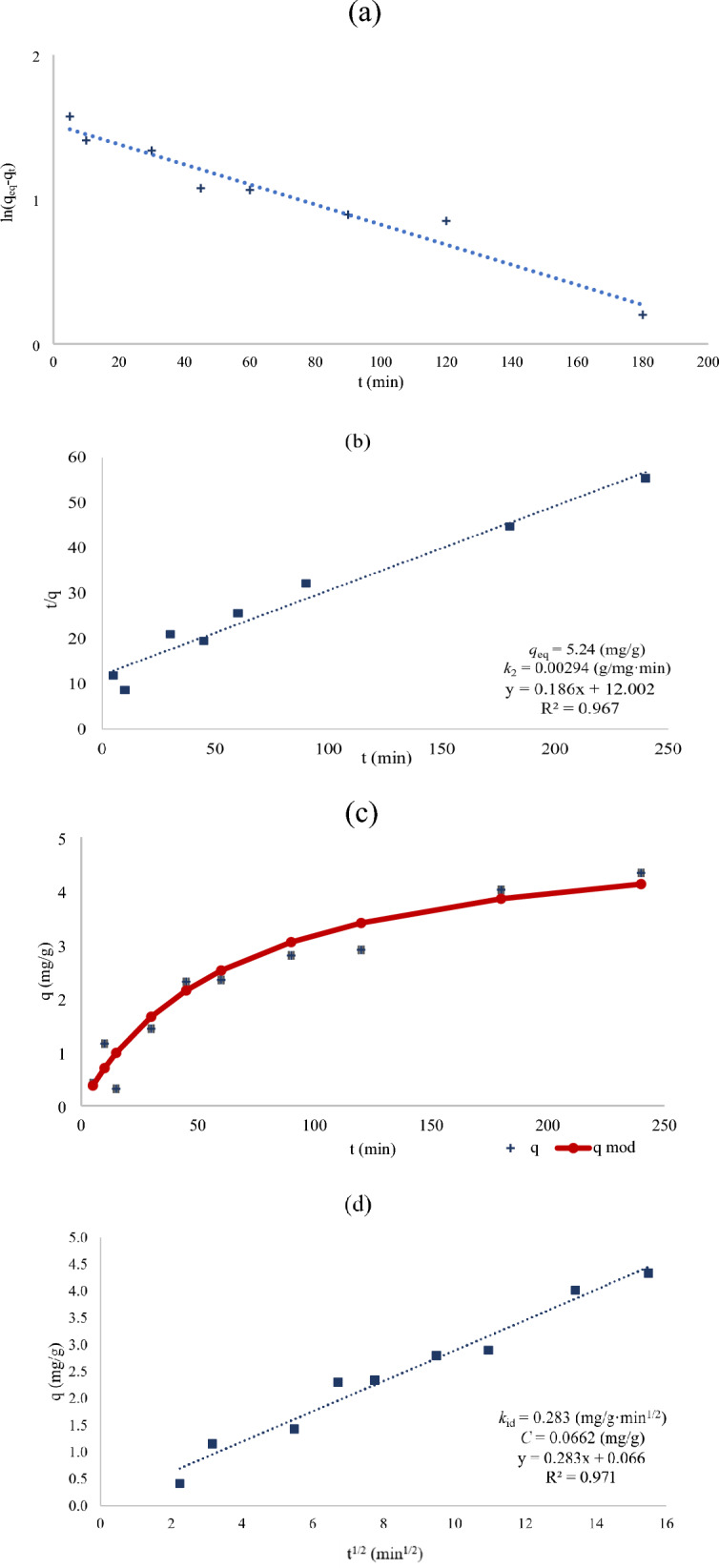
Linearization of pseudo-first-order model (**a**), linearization of pseudo-second-order model (**b**), pseudo-second-order kinetic model (**c**) and the intramolecular diffusion model Weber–Morris (**d**) for sorption of phosphate ions by modified biochar obtained at 700 °C

The results are shown in Fig. 2, which presents the calculated parameters and graphs of the linearization of PFO and PSO, the kinetic model of PSO, and the Weber–Morris model.

The kinetics of solid–liquid system was described by the PFO and PSO models, which were based on physical and chemical adsorption, respectively (Eduah et al. 2020). As for the sorption of phosphate ions, the higher value of the coefficient of determination was recorded for the PSO model than PFO model, which was responsible for the occurrence of chemisorption. Therefore, after presenting the linearization of both models, the kinetic model is only shown for PSO (Eduah et al. 2020; Zhou et al. 2019).

To have a deeper insight into the mechanism of phosphate anion adsorption the intramolecular model was applied. The Weber–Morris plot may show multi-linearity, indicating that there are several steps in the adsorption mechanism. The uptake rate can be limited by the size of the adsorbate molecules, the concentration of the adsorbate, the diffusion coefficient of the adsorbate in the bulk phase and in the pores of the adsorbent, the affinity towards the adsorbent and the degree of mixing. Figure 2d shows the plot of fractional uptake. It can be observed that the plot is almost linear over the entire time range, implying that one process affects the adsorption of phosphates on the biochar. It is usually said that if intraparticle diffusion is the only rate-controlling step, then the plot will pass through the origin. Otherwise, the boundary layer controls the adsorption to some extent (Özcan and Özcan 2004). The presence of boundary layer diffusion in this system can be deduced from the fact that the plot does not pass through the origin. However, the thickness of the boundary layer is small, *C* = 0.0662, indicating the previously mentioned high affinity of the phosphate anion to the adsorbent surface.

For adsorbents with developed porosity, such as activated carbons, intraparticle diffusion usually occurs in two stages. The adsorbate molecules rapidly enter transport pores (macropores and wider mesopores), described by the first part of the graph, and then more slowly enter smaller pores where final adsorption takes place, depicted by the second part of the graph. Here, we can only see one line. This is because biochar has not developed microporosity. This means that adsorption occurs on the surface of the macropores.

Comparing our results with those reported by the literature (Table 4), it can be seen that the parameters of pyrolysis such as temperature, type of biomass used to produced biochar, duration time of the pyrolysis and the sorption process, such as pH and initial concentration of phosphate ions, as well as the sorbent content in the solution, had a large impact on sorption capacity. For three works cited (Jiang et al. 2019; Qiu et al. 2019; Zheng et al. 2020), the values of sorption capacity towards phosphate ions calculated from PFO and PSO were the most similar to those obtained in this study. Jiang et al. (2019) and Zheng et al. (2020) modified biomass with magnesium ions. Jiang et al. (2019) analyzed six different biomasses from which biochar was produced. Compared to this research, similar values for the sorption capacity of phosphate ions (determined from the PFO and PSO models) were obtained by Jiang et al. (2019) and were equal to 6.92 and 7.46 mg/g, respectively, for the raw material *Camellia oleifera*. Biochar produced from *Broussonetia papyrifera* leaves also produced a similar sorption capacity for phosphate ions (6.68 and 7.61 mg/g for the PFO and PSO models), although under quite different process pyrolysis and sorption conditions (Qiu et al. 2019).

#### Equilibrium of sorption process of phosphate ions

Of the many different equations used in the literature to interpret equilibrium adsorption isotherms, the most popular are the Langmuir [Eq. (8)] and Freundlich [Eq. (10)] models, which have been used to interpret the data. The results of the parameters calculated for the Freundlich and Langmuir isotherms for the sorption of phosphate ions are shown in Table 5. According to the calculated dimensionless separation factor *R*_L_, the adsorption process was favorable (0 < *R*_L_ < 1) for each content of the sorbent in the solution (1 g/L and 10 g/L). All values of 1/*n* were lower than 1, which means that modified biochar preferably removed phosphate ions. The same was observed by Agrafioti et al. (2014) for the sorption of arsenic and chromium ions by biochar produced from solid waste at 300 °C. The Langmuir isotherm better described the adsorption of the phosphate ions by modified biochar than the Freundlich isotherm due to the higher value of the coefficient of determination. Therefore, the values of the Langmuir isotherm were chosen, contrary to what is commonly practiced (Table 6).

**Table 5:** Literature research on the sorption of phosphate ions by various biosorbents

Raw material—biochar	Pyrolysis conditions	Sorption conditions	Biosorption kinetics*		Biosorption equilibrium**	References
			Pseudo-first-order model	Pseudo-second-order model	Langmuir isotherm	
			*q* (mg/g)			
Banana straw	Modified with MgCl_2_, 430 °C for 4 h	*C*_s_ 5 g/L *C*_0_ 250 mg/L* *C*_0_ 20–350 mg/L** pH (established, not reported) 4 h	41.3	49.3	31.2	Jiang et al. (2019)
Cassava straw			13.3	15.3	31.1	
Chinese fir straw			11.9	12.6	6.77	
Corn straw			10.2	10.1	12.3	
Taro straw			13.5	14.2	14.4	
*Camellia oleifera* shell			6.92	7.46	17.0	
*Broussonetia papyrifera* leaves	500 °C for 2 h	*C*_s_ 5 g/L *C*_0_ 50 mg/L* *C*_0_ 0–80 mg/L** pH 7.0 4 h	6.68	7.61	35.6 (for 25 °C)	Qiu et al. (2019)
Corn straw	Modified with Fe(NO_3_)_3_9H_2_O, 600 °C for 2 h	*C*_s_ 2 g/L *C*_0_ 35 mg/L* *C*_0_ 17.5–1400 mg/L** pH 5.28 192 h	10.3	10.9	57.4	Zhang et al. (2021)
Wheat straw	Modified with MgCl_2_–AlCl_3_, 600 °C for 2 h	*C*_s_ 7 g/L *C*_0_ 100 mg/L* *C*_0_ 0–2000 mg/L** pH 6.0 24 h	13.1	13.6	153	Zheng et al. (2020)
	Modified with MgCl_2_, 600 °C for 2 h		2.86	3.63	9.64	
	Modified with AlCl_3_, 600 °C for 2 h		8.70	8.98	82.8	
	600 °C for 2 h		1.46	1.58	1.64	
Rice straw	700 °C for 2 h	*C*_s_ 10 g/L *C*_0_ 20 mg/L* *C*_0_ 0–320 mg/L** pH 7.0 24 h	0.998	1.79	5.41	Zhou et al. (2019)
*Phragmites communis*	300 °C for 2 h		0.430	0.084	7.75	
Sawdust	300 °C for 2 h		0.100	0.024	3.86	
Egg shell	300 °C for 2 h		1.88	0.827	4.54	
	500 °C for 2 h		2.03	1.51	4.92	
	700 °C for 2 h		1.53	1.03	6.08	
Raw sludge	Modified with Fenton reagent (110 mg Fe(II)/g and 88 mg H_2_O_2_/g), 300 °C for 2 h	*C*_s_ 15 g/L *C*_0_ 46 mg/L* *C*_0_ 23–161 mg/L** pH 7.0 52 h	1.94	2.97	2.64	Wang et al. (2020a, b)
Carrot residues	Modified with MgCl_2_H_2_O, 400 °C for 4 h	*C*_s_ 0.5 g/L *C*_0_ 30 mg/L* *C*_0_ 25–350 mg/L** pH 8.0 36 h	30	16	138	Pinto et al. (2019)
Seaweed *Ulva intestinalis*	Modified with MgCl_2_6H_2_O, 700 °C for 1 h	*C*_s_ 1 g/L *C*_0_ 100 mg/L* *C*_0_ 5–100 mg/L** pH 5.0 4 h	4.70	5.24	5.92	This work

*C*_s_ sorbent content in the solution; C_0_ initial concentration of phosphate ions; ^*^ Biosorption kinetics; ^**^ Biosorption equilibrium

**Table 6:** Langmuir and Freundlich isotherms for the sorption of phosphate ions by B700MgCl_2_

Isotherm	Conditions	Parameter
Langmuir	pH 5, *C*_0_ 0–100 mg/L, *C*_s_ 1 g/L	*q*_max_ 5.92 mg/g *b* 0.0111 L/mg *R*_L_ 0.796 *R*^2^ 0.960
	pH 5, *C*_0_ 0–100 mg/L, *C*_s_ 10 g/L	*q*_max_ 12.0 mg/g *b* 0.271 L/mg *R*_L_ 0.0387 *R*^2^ 0.905
Freundlich	pH 5, *C*_0_ 0–100 mg/L, *C*_s_ 1 g/L	*n* 1.74 *k*_F_ 0.288 *R*^2^ 0.476
	pH 5, *C*_0_ 0–100 mg/L, *C*_s_ 10 g/L	*n* 2.60 *k*_F_ 2.65 *R*^2^ 0.747

For the sorbent content in the solution equal to 1 g/L, the value of the maximum sorption capacity of phosphate ions by modified biochar was 5.92 mg/g. Zhou et al. (2019) presented a very similar value of maximum sorption capacity (6.08 mg/g) for the binding of phosphate ions by biochar produced from eggshells. The conditions of the sorption process (*C*_s_ 10 g/L, *C*_0_ 0–320 mg/L, pH 7, 24 h) were significantly different compared to those used in this study. When comparing the values for the sorbent content in a solution—10 g/L—the maximum sorption capacity in this research was 12.0 mg/g. In a study by Jiang et al. (2019), the maximum sorption capacity of biochar (made from corn straw) with respect to phosphate ions was 12.3 mg/g (*C*_s_ 5 g/L, *C*_0_ 20–350 mg/L, 4 h) and was very close to the value determined in this study. Jiang et al. (2019) also modified biomass (corn straw) with magnesium ions before pyrolysis. However, it must be emphasized that comparison of removal efficiency is difficult as many variables such as porosity, adsorbent dosage, pH of the solution, etc. have to be taken into account.

In the next step, the sorption of phosphate ions was carried out for B700 and B700MgCl_2_ for their content of 10 g/L in the solution. The aim of this test was to compare the sorption capacity of the two adsorbents with similar S_BET_ and, in addition, to produce phosphorus-enriched biochar to be used in later tests on plants. This solution was prepared from potassium (I) dihydrogen phosphate and contained 98.5 mg/L of P and 117 mg/L of K (confirmed by ICP–OES analysis). Multielemental analysis of the solution was performed before and after biosorption process. A higher concentration of P occurred in the solution after the biosorption process for biochar produced at 700 °C. This is confirmed by the fact that modified biochar adsorbs phosphate ions better than pristine biochar does. When using modified biochar, P was adsorbed from synthetic sewage in 84.3%, which is twice as much compared to 40.6% with B700 determined by the ICP–OES technique. Considering the same sorption condition and S_BET_ value, the higher removal on the MgCl_2_ modified biochar compared to the unmodified biochar can be explained by the biochar porosity and composition. For modified biochar, the Ca and Mg concentration in the solution was higher (20.6 and 270 mg/L) than for B700 (0.749 and 4.24 mg/L). Unlike other elements, such as potassium and sodium, their contents in B700 and B700MgCl_2_ were almost equal, with 309 (K) and 109 (Na) mg/L for B700, and 244 (K) and 108 (Na) mg/L for B700MgCl_2_.

#### FTIR

**Fig. 3:**
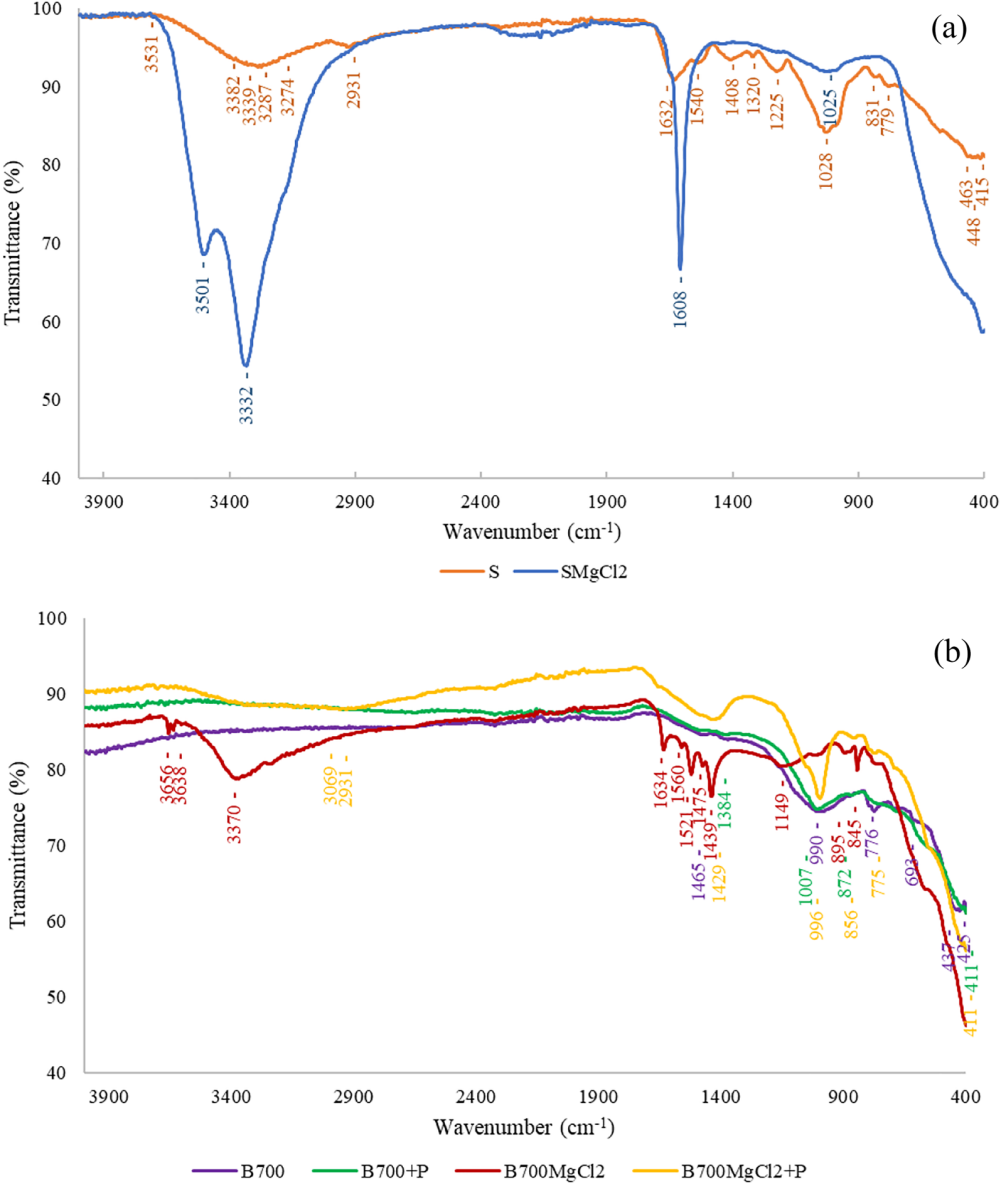
FTIR spectra for (**a**) seaweed *Ulva intestinalis* (S) and modified *Ulva intestinalis* (SMgCl_2_), (**b**) biochar obtained at 700 °C (B700), biochar obtained at 700 °C enriched with phosphate ions (B700 + P), modified biochar produced at 700 °C (B700MgCl_2_), and modified biochar obtained at 700 °C enriched with phosphate ions (B700MgCl_2_ + P)

FTIR spectra were obtained for seaweed and seaweed-derived bioproducts as shown in Fig. 3 and the corresponding peaks with the assignment of functional groups in Table 7. The hydroxyl groups (–OH) in the range 3100–3600 cm^−1^ were observed in S, SMgCl_2_, B700MgCl_2_, and B700MgCl_2_ + P. In one cited work (Vijayaraghavan and Balasubramanian 2021) a similar FTIR analysis of biochar produced from pine waste was carried out. The vibrations for aromatic groups occurred at 620–860 cm^−1^. In this study, no such peaks (in the range 620–860 cm^−1^) were observed for the spectrum of modified *Ulva intestinalis*. For the biochar enriched with phosphate ions, one peak at 872 cm^−1^ was observed and for the remaining tested sorbents two vibrations for the aromatic group were visible on the spectrum. The phosphate group (P–OH, P=O) was identified for each analyzed sample in the range of 990–1150 cm^−1^. For some of the sorbent (S, B700, B700 + P, B700MgCl_2_ + P), the peak was more stretched, which was further confirmed by the ICP–OES analysis with a higher phosphorus content in these samples. The same was also reported in the article by Nardis et al. (2021). In the spectra of seaweed and modified biochar obtained at 700 °C enriched with phosphate ions, the vibration at 2931 cm^−1^, related to the aliphatic group (-CH_2_) was observed. It was the same as in the work of Eduah et al. (2020), who examined biochar produced from cocoa pod husk, corn cob, rice husk, and palm kernel shell pyrolyzed at 300 °C and 650 °C. Eduah et al. (2020) noted that the carboxyl group can be seen in the range of 1300–1700 cm^−1^. Satisfactorily, as can be seen in Fig. 3, -COOH group was observed to vibrate at 1465 cm^−1^ (B700), 1384 cm^−1^ (B700 + P), 1608 cm^−1^ (SMgCl_2_), 1429 cm^−1^ (B700MgCl_2_ + P). More vibrations were observed in the range of 1225–1634 cm^−1^ (S and B700MgCl_2_), which could suggest the presence of other groups, such as C–C, C–H, C–O, and –COOH (Vijayaraghavan and Balasubramanian 2021).

**Table 7:** Assigned peaks visible in the FTIR spectra for seaweed and seaweed-derived bioproducts

Raw material/biochar	FTIR range	Peak	Chemical compound groups
Seaweed	3100–3600	3531	Hydroxyl groups
		3382	
		3339	
		3287	
		3274	
	2900–2950	2931	Aliphatic groups
	1300–1700	1632	Carboxyl groups
		1540	
		1408	
		1320	
		1225	
	990–1150	1028	Phosphate groups
	620–900	831	Aromatic groups
		779	
	< 600	463	Alkyl halides groups
		448	
		415	
Modified seaweed	3100–3600	3501	Hydroxyl groups
		3332	
	1300–1700	1608	Carboxyl groups
	990–1150	1025	Phosphate groups
Biochar obtained at 700 °C	1300–1700	1465	Carboxyl groups
	990–1150	990	Phosphate groups
	620–900	776	Aromatic groups
		693	
	< 600	437	Alkyl halides groups
		425	
Biochar obtained at 700 °C enriched with phosphate ions	1300–1700	1384	Carboxyl groups
	990–1150	1007	Phosphate groups
	620–900	872	Aromatic groups
	< 600	411	Alkyl halides groups
Modified biochar obtained at 700 °C	3100–3600	3656	Hydroxyl groups
		3638	
		3370	
	1300–1700	1634	Carboxyl groups
		1560	
		1521	
		1475	
		1439	
	990–1150	1149	Phosphate groups
	620–900	895	Aromatic groups
		845	
Modified biochar obtained at 700 °C enriched with phosphate ions	3100–3600	3069	Hydroxyl groups
	2900–2950	2931	Aliphatic groups
	1300–1700	1429	Carboxyl groups
	990–1150	996	Phosphate groups
	620–900	856	Aromatic groups
		775	
	< 600	411	Alkyl halides groups

#### SEM–EDS

Table 8 shows the range of percentage content of elements in the surface layer of the examined raw materials/biochar, which reflects EDS analysis. The results presented below are different from the data in Table 3. The discrepancies in the measurement data (Tables 3 and 8) results from the use of different analytical methods. The possibility of detecting individual elements and their quantitative assessment depends on the measurement method. In the ICP–OES analysis, the elemental content is related to the entire sample volume (Table 3), while the measurement data from the EDS analyses (Table 8) come from the surface layer of the tested material. In the surface layer of dried algae (sample S) there are a number of elements (Table 8), mainly C (35.4–39.3 wt.%) and O (ca. 40 wt.%). In the dried biomass, there are also other elements, e.g., Ca, K, Na, Si, S. These elements are part of the mineral components in this material. Despite heat treatment (pyrolysis at 700 °C) and enrichment of biochar (in MgCl_2_ or phosphate ions), no significant changes occurred in the content of, for example, Ca, K, Na, Si, S in the surface layer of the tested material (Table 8). It is similar in the case of chromium, manganese and iron: the content of these elements in the surface layer of individual samples shows a constant value (below 1 wt.%) regardless of the biomass modification (Table 8). A separate group of elements are Mg, Cl and P (Table 8). These are the elements that take part in biomass modification or enrichment. Changes in the content of Mg and P inform about the processes that occur on the surface of the material after individual stages of modification or enrichment. In the surface layer of dried biomass, the content of Mg and P does not exceed (respectively) 5 wt.% and 1.5 wt.%. Pyrolysis of *Ulva intestinalis* (sample B700) causes decomposition of organic substances in the alga and its partial dehydration. As a result, the content of C, Mg and P in biochar increases, while the amount of O and Cl remains constant (Table 8). Such modified biochar in an aqueous solution containing phosphate ions (samples B700 + P) results in an approx. twofold increase in the content of P and O (Table 8), which confirms the adsorption of PO_4_^3−^ ions on the surface of this material. Dried algae in a solution containing MgCl_2_ (samples SMgCl_2_) results in a Four to fivefold increase in the Mg content on the surface of *Ulva intestinalis* (Table 8). The presence of MgCl_2_ on the surface of the sample also results in an increase in the Cl content. Such a modification of biomass causes a decrease in the content of other elements in the surface layer of the SMgCl_2_ sample in comparison to the dried algae (Table 8). Pyrolysis of biomass modified with MgCl_2_ (samples B700MgCl_2_) results in thermal dissociation of this compound, which leads (above 500 °C) to the formation of, e.g., MgO. After exposure to a solution containing phosphate ions on the surface of Mg modified biochar (samples B700MgCl_2_ + P), an approximately threefold increase in P content was found compared to sample B700MgCl_2_ (Table 8), which confirms the sorption of PO_4_^3−^ ions on the surface of modified biomass. The data in Table 8 show that the modification of biomass in MgCl_2_ before its pyrolysis improves the sorption of phosphate ions on the surface modified in this way.

**Table 8:** Summary of the percentage by weight percent of elements for the analyzed raw materials/biochar (SEM–EDS)

Weight percent (%; range)						
Elements	S	B700	B700 + P	SMgCl_2_	B700MgCl_2_	B700MgCl_2_ + P
C	35.4–39.3	37.3–46.3	27.2–48.5	9.2–14.1	8.7–14.4	14.7–21.5
O	40.2–40.5	27.7–31.9	27.4–34.5	17.9–23.1	24.1–36.9	34.1–36.3
Mg	4.3–4.7	5.5–8.9	6.4–6.9	17.7–23.8	16.1–35.3	27.3–37.4
Cl	0.8–1.8	0.5–1.7	0.06–0.09	33.1–47.3	9.6–14.4	0.4–0.5
P	0.7–1.3	0.6–2.3	1.4–3.1	0.3–0.9	0.4–0.6	1.5–1.9
Ca	0.9–1.9	2.1–4.7	2.9–5.8	0.1–0.3	0.7–1.6	0.3–0.5
K	2.5–4.8	2.4–4.6	1.9–3.2	1.2–1.4	0.4–0.8	0.1–0.2
Na	2.3–4.5	6.3–6.6	1.8–3.2	0.4–1.5	0.9–2.3	0.3–0.7
Si	0.5–1.1	1.1–3.7	0.8–3.1	0.3–0.9	0.2–1.6	0,5–0.8
S	4.8–7.4	2.7–8.9	5.5–9.1	0.4–1.2	0.6–2.9	0.4–1.1
Cr, Mn, Fe	0.14–0.45	0.4–0.7	0.5–0.9	0.2–0.8	0.4–0.7	0.3–0.6

**Fig. 4:**
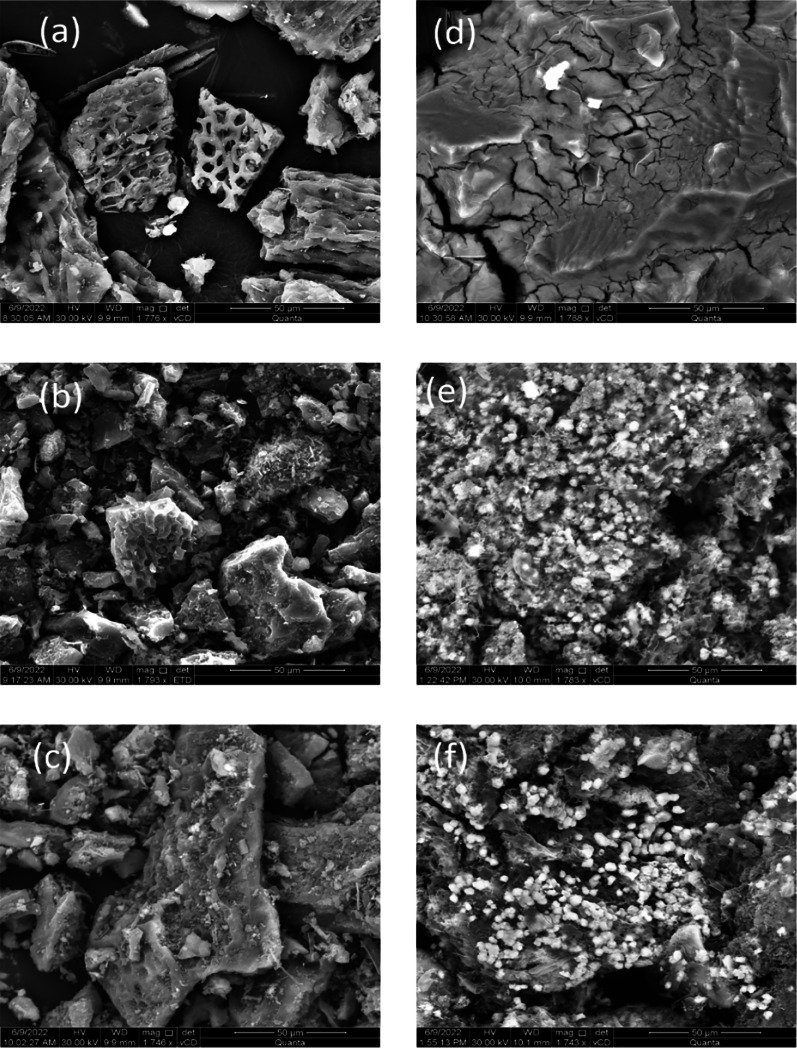
Presentation of the surface of tested sorbents using SEM (scale 50 μm): (**a**) seaweed *Ulva intestinalis*, (**b**) biochar obtained at 700 °C, (**c**) biochar obtained at 700 °C enriched with phosphate ions, (**d**) modified *Ulva intestinalis*, (**e**) modified biochar produced at 700 °C, (**f**) modified biochar obtained at 700 °C enriched with phosphate ions

Figure 4 shows changes in the surface topography (SEM images) of *Ulva intestinalis* algae after various stages of its modification. The dried biomass material is characterized by high surface irregularity and porosity (Fig. 4a). Pyrolysis of seaweed (at 700 °C) results material fragmentation and reduction in surface unevenness (Fig. 4b). Enrichment of biochar produced at 700 °C (sample B700 + P) with phosphate ions does not cause significant changes in the topography of this material compared to the treatment of seaweed subjected only to pyrolysis (Fig. 4c). Enrichment of *Ulva intestinalis* algae with MgCl_2_ (samples SMgCl_2_) results in a decrease in seaweed surface porosity (Fig. 4d). Visible cracks on the surface of this sample indicate that MgCl_2_ was deposited on the seaweed in a thick layer. The characteristic irregularities that were observed on the unmodified surface of the alga are not visible (Fig. 4a, d). As a result of pyrolysis (at the temperature of 700 °C) on the surface of biochar enriched with MgCl_2_ (samples B700MgCl_2_), numerous fine precipitates (several micrometers in size) of irregular shape are formed (Fig. 4e). As a result of the enrichment of this material with phosphate ions (samples B700MgCl_2_ + P), precipitations are also observed on the surface of this sample with a more regular shape compared to the B700MgCl_2_ sample (Fig. 4e).

### Use in agriculture

#### Phytotoxkit tests

**Fig. 5:**
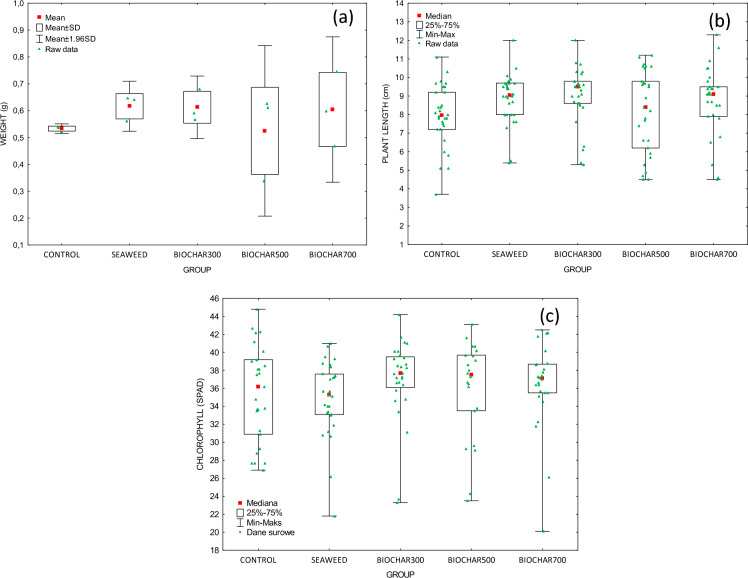
Results of the phytotoxkit tests (**a**) fresh weight of the aboveground parts of plants, (**b**) plant length, (**c**) chlorophyl (SPAD) using different soil additives

To verify the potential toxicity of seaweed and seaweed-derived biochars, phytotoxkit tests were performed on wheat (*Triticum aestivum*). Table 9 and Fig. 5 summarize the results of the measurements of chlorophyll content, plant length, and its fresh weight after the tests and soil pH. For all measured parameters, no statistically significant differences were found between the examined groups. However, the addition of the soil additive (seaweed and biochar) allowed a higher plant growth compared to the control group. When comparing the results for the length of above-ground parts of plants, the best results were obtained in the group using biochar produced at 300 °C. However, the measured plant length was higher for each soil additive than in the control group. Chlorophyll content in the leaves was the highest for all samples with the biochar added to the soil. In the case of the fresh weight of the aboveground parts of plants, the highest value was achieved in the group where seaweed was added. When biochar is added to the soil, its alkalinity increases. Moreover, alkalinity rises with the increase in the temperature of biochar production; all pH values for the biochar groups are higher than for the control group (Nardis et al. 2021). As described above, pH of the biochar increased with the increase in pyrolysis temperature. The phytotoxkit tests confirmed that none of the soil additives had a toxic effect on the plant. Kraska et al. (2016) examined the toxicity of biochar using commercial toxicity bioassay—Phytotoxkit (2004). In the work cited, biochar was produced from wheat straw. The results revealed that this biochar was also not toxic to plants (*Lepidium sativum* L.) due to better root growth of plants in the group with the addition of biochar (Kraska et al. 2016).

**Table 9:** Phytotoxkit tests seaweed and seaweed-derived biochars used as soil additives

Group	Fresh plant weight**	Plant length*	Chlorophyll (SPAD)*	pH of soil
	Mean ± SD (g)	Median (cm)	Median (–)	Mean ± SD (–)
C	0.533 ± 0.009	7.95	36.2	5.52 ± 0.09
S	0.617 ± 0.047	9.05	35.3	5.71 ± 0.10
B300	0.613 ± 0.059	9.50	37.7	5.69 ± 0.02
B500	0.525 ± 0.162	8.40	37.5	5.72 ± 0.04
B700	0.604 ± 0.138	9.10	37.1	6.06 ± 0.13

*C* control sample; ^*^Kruskal-Wallis test; ^**^ Tukey multiple comparison test

#### Pot experiments using enriched biochar

**Fig. 6:**
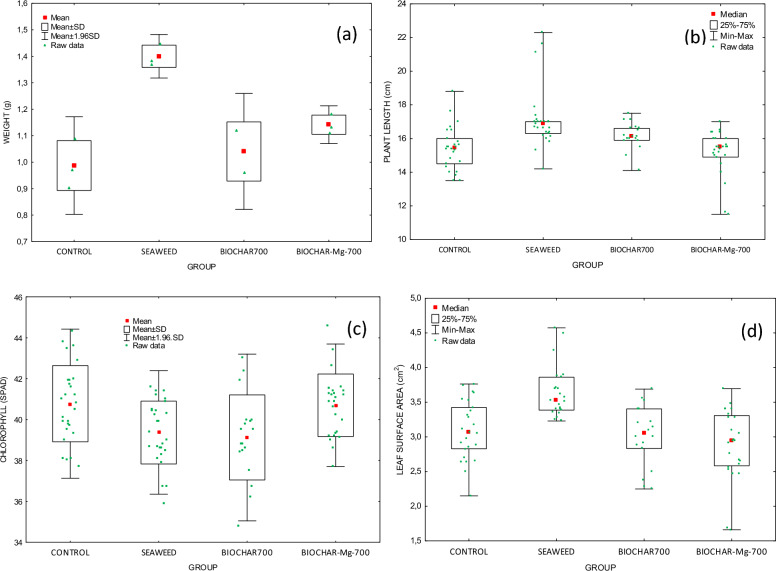
Results of the pot experiments (**a**) fresh weight of the aboveground parts of plants, (**b**) plant length, (**c**) chlorophyl, (**d**) leaf surface area using different soil additives

Pot experiments carried out after phytotoxicity studies on the same plant showed that biochar was not toxic to wheat (*Triticum aestivum*). Three different soil additives were used for these tests—*Ulva intestinalis* (S), biochar enriched with phosphate ions (B700 + P) and modified biochar enriched phosphate ions (B700MgCl_2_ + P). The results are presented in Table 10 and Fig. 6.

**Table 10:** Pot experiments for seaweed and seaweed-derived biochars used as soil additives

Group	Plant fresh weight	Plant length	Chlorophyll (SPAD)	Leaf surface area	pH of soil
	Mean ± SD** (g)	Median* (cm)	Mean ± SD** (–)	Median* (cm^2^)	Mean ± SD (–)
C	0.987 ± 0.094^a^	15.5^a^	40.8 ± 1.9^a,b^	3.06^a^	6.03 ± 0.08
S	1.40 ± 0.04^a,b,c^	16.9^a,b^	39.4 ± 1.5^a,c^	3.53^a,b,c^	6.14 ± 0.09
B700 + P	1.04 ± 0.11^b^	16.1^c^	39.1 ± 2.1^b,d^	3.05^b^	6.78 ± 0.08
B700MgCl_2_ + P	1.14 ± 0.04^c^	15.5^b,c^	40.7 ± 1.5^c,d^	2.94^c^	8.95 ± 0.08

*C* control sample; ^*^Kruskal-Wallis test; ^**^ Tukey multiple comparison test; ^a,b,c^ Statistically significant differences (*p* < 0.05)

Due to the porous structure and large specific surface area, biochar has the ability to retain water and nutrients in the soil (Olmo et al. 2014). This stimulates plant growth; therefore, plants cultivated in soil containing these soil additives had higher values of the length and fresh weight of the aboveground part. The highest value of the fresh weight of the aboveground part was displayed by seaweed; however, for the remaining soil additives, the value was higher compared to the control sample. In comparison to biochars, higher values of plant fresh weight and length were brought about by algae as a soil additive. This may be caused by too short a time of residence of biochar in the soil before pot tests and too short duration of pot tests. In addition, algae decompose much faster in the soil, providing nutrients for plants, while biochar is much more stable in the soil (Budai et al. 2013). A better effect of using enriched biochar with phosphorus on plant growth occurred for the longer duration of pot tests, e.g.: 21 days (Nardis et al. 2021), 65 days (Gunes et al. 2014), 120 days (Ng et al. 2022), which suggests conducting longer pot tests in the future using biochar as a soil additive. There were statistically significant differences for measuring the weight of fresh plants, the length of the plants, chlorophyll, and the leaf surface area for different groups, when comparing in a column (marked in Table 8). The chlorophyll content (SPAD meter) measurements showed that the highest median value was for the group treated with B700MgCl_2_ + P. Soil enriched with B700MgCl_2_ + P had the highest pH after completing the pot experiments. For the remaining groups, the soil pH was similar to each other and was approximately 6. Pot experiments were carried out on wheat *Triticum aestivum*, which grows well in soil with a pH between 6.1 and 7.5 (Cherlinka 2016). The addition of biochar enriched with phosphorus increases the soil pH, which is beneficial for this variety of wheat. In the article by Nardis et al. (2021), pot experiments were performed with three different soil additives (modified Mg P-loaded biochars produced from poultry litter, pig manure, and sewage sludge). All soil additives led to higher values amounts of shoot dry matter, length plant growth, phosphorus, and magnesium compared to the control sample. In addition, they allowed the plant to be enriched with valuable ingredients, such as Mg and P. Magnesium chloride was selected for biochar modification, because magnesium is a secondary macronutrient that is necessary for proper plant growth, e.g.: supports root growth, increase crop yield, is necessary for the photosynthesis process, and protects it from stress in unfavourable conditions (Wang et al. 2020a, b). A plant enriched with biochar containing phosphorus grows better and develop more properly. All this suggests that biochar can be a good method of recovering phosphorus from wastewater while biochar enriched with phosphate ions can be used in agriculture as a soil additive, supplementing this important macronutrient.

## Conclusion

In this work, the potential of the green macroalgae *Ulva intestinalis* was shown in the production of precious bioproducts. Three biochars were produced from the biomass of this seaweed by pyrolysis at 300, 500, and 700 °C. The examination of the porous structure and specific surface area of the bioproducts, led to the discovery that biochar produced at 700 °C generated the highest results. Due to the insufficient adsorption of phosphate ions by B700, seaweed was modified before pyrolysis with magnesium chloride. Changing the properties of biomass by modifying it and subjecting it to the pyrolysis process allows to obtaining biochar, which facilitates the removal of phosphate ions, made it possible to carry out the kinetics and equilibrium of the sorption process. Since biochar is non-toxic and enhances plant development, it could be applied as a soil additive in conformity with the concept of circular economy. A way to use the waste generated in the wastewater treatment process is to remove the phosphate ions from the wastewater and then utilize biochar enriched with phosphate ions as soil additive.

## Data Availability

The data sets used or analyzed during the current study are available from the corresponding author on request.
